# Neuropsychological evidence for the temporal dynamics of category-specific naming

**DOI:** 10.1080/13506285.2017.1330790

**Published:** 2017-06-06

**Authors:** Sven Panis, Katrien Torfs, Celine R. Gillebert, Johan Wagemans, Glyn W. Humphreys

**Affiliations:** ^a^ Experimental Psychology Unit, University of Kaiserslautern, Kaiserslautern, Germany; ^b^ Laboratory of Experimental Psychology, University of Leuven, Leuven, Belgium; ^c^ Department of Experimental Psychology, University of Oxford, Oxford, UK

**Keywords:** Object recognition, category-specific impairments, event history analysis

## Abstract

Multiple accounts have been proposed to explain category-specific recognition impairments. Some suggest that category-specific deficits may be caused by a deficit in recurrent processing between the levels of a hierarchically organized visual object recognition system. Here, we tested predictions of interactive processing theories on the emergence of category-selective naming deficits in neurologically intact observers and in patient GA, a single case showing a category-specific impairment for natural objects after a herpes simplex encephalitis infection. Fragmented object outlines were repeatedly presented until correct naming occurred (maximum 10 times), and the fragments increased in length with every repetition. We studied how shape complexity, object category, and fragment curvature influence the timing of correct object identification. The results of a survival analysis are consistent with the idea that deficits in recurrent processing between low- and high-level visual object representations can cause category-selective impairments.

A striking observation is that certain brain-damaged patients can successfully recognize exemplars of some object categories (tools, body parts, etc.) but not others. Since the experimental report of category-specific deficits by Warrington and Shallice (1984), numerous other cases have been described in different patient populations (e.g., Barbarotto, Capitani, & Laiacona, 1996; Blundo, Ricci, & Miller, 2006; Burnett, Panis, Wagemans, & Jellema, 2015; Humphreys & Riddoch, 2003; Lambon Ralph, Lowe, & Rogers, 2007; Sartori, Job, Miozzo, Zago, & Marchiori, 1993; Tsapkini, Frangakis, & Hillis, 2011; Warrington & McCarthy, 1987). Most commonly, reports concern patients with a category-specific impairment for natural objects, but there have also been a few reports of patients with impairments for manmade or artefactual objects (see Caramazza, 1998; Forde & Humphreys, 1999; Humphreys & Forde, 2001; Tyler & Moss, 2001, for reviews).

## Theories of category-specific naming deficits

Several accounts have been proposed to explain such category-specific recognition impairments. The sensory/functional account was proposed by Warrington and Shallice (1984). They investigated the visual identification deficits of four patients who made a partial recovery from a herpes simplex encephalitis (HSE) infection. The patients showed a selective impairment in visual identification and verbal comprehension of living things and foods, but not of inanimate objects. Warrington and Shallice suggested that distinguishing living things and foods critically depends upon fine differences in colour, shape, size, and texture. In contrast, inanimate objects have specific functions, and are designed for activities appropriate for their function. The authors proposed two semantic systems: one storing sensory information and the other functional, use-related information. Accordingly, disruption of the sensory semantic system will lead to deficits in recognizing natural objects, whereas disruption of the functional semantic system will lead to deficits in recognizing manmade objects.

The domain-specific account was proposed by Caramazza and Shelton (1998), who argued that dissociations between different types of object categories arise because semantic knowledge is organized in a domain-specific manner, distinguishing natural and manmade stimuli. They argued that “evolutionary pressures have resulted in specialized mechanisms for perceptually and conceptually distinguishing animate and inanimate kinds leading to a categorical organization of this knowledge in the brain” (p. 17).

The unitary system account states that concepts are represented as patterns of activation over multiple semantic properties within a unitary distributed system (Caramazza, Hillis, Rapp, & Romani, 1990; Devlin, Russell, et al., 2002; Tyler & Moss, 2001). Category-specific deficits emerge after focal damage to the semantic system because concepts differ in their structure and content (i.e., the correlations between sensory and functional attributes, and associations between the shape of visual parts and their functions), and not because conceptual knowledge is divided in separate stores. This account emphasizes the role played by the extent and severity of brain damage rather than its precise location.

Although these accounts make different assumptions about the extent and the localization of brain lesions resulting in category-specific disorders, it is becoming clear that there is a correlation between the locus of the lesion and the pattern of categorical impairment (Devlin, Moore, et al., 2002; Gainotti, 2000; Gainotti, Silveri, Daniele, & Giustolisi, 1995). Patients with a category-specific (semantic, not purely visual or lexical) impairment for living objects (mainly due to HSE, and not head trauma or semantic dementia) typically show a bilateral injury to the anterior and inferior parts of the temporal lobes (the temporal pole, the inferior temporal [IT] cortex, etc.). Patients with a category-specific impairment for manmade objects typically show a lesion in fronto-parietal areas of the left hemisphere (e.g., the left fronto-temporo-parietal area). Furthermore, the category body-parts was typically spared in the former patients but impaired in the latter, while the category food was typically spared in the latter patients but impaired in the former (Gainotti, 2000).

These results show that the pattern of categorical impairment does not respect the natural/living versus manmade/nonliving distinction, and strongly suggest that the injured brain areas house exactly those neurophysiological mechanisms that contribute to the *acquisition* of the disrupted semantic categories: the acquisition account (Gainotti et al., 1995). For example, the anterior temporal lobes receive convergent, integrated input from visual, auditory, olfactory, and gustatory sensory systems (Blaizot et al., 2010), and the anterior IT cortex represents high-level (structured) visual representations useful to discriminate structurally similar members. Semantic knowledge about natural objects and food is thus mainly based on high-level visual processing and integration of sensory information. In contrast, somato-sensory and motor memories are mainly represented in the fronto-parietal areas, and knowledge about (small manipulable) manmade objects, furniture, and body-parts is mainly based on memories about handling, manual use, or physical contact.

Nevertheless, some authors have argued that the observed heterogeneity in category-specific deficits will only be understood completely when taking into account the *dynamics* of normal object recognition processes during naming performance (Gerlach, 2009; Humphreys & Forde, 2001; Humphreys, Riddoch, & Quinlan, 1988; Sartori & Job, 1988). For example, to explain that healthy observers name intact line-drawings of manmade objects faster compared to natural objects, Humphreys et al. (1988) suggested that normal recognition consists of three stages, each entailing access to specific memories. First, structural descriptions contain information about global shape and the configuration of parts. Second, semantic representations include information about an object’s use and its associations with other objects. Third, lexical and phonological representations contain information about the different abstract labels that can apply to the input. Humphreys et al. (1988) additionally assumed: (1) that processing operates in cascade (i.e., activation can be passed on to the next stage before processing at an earlier stage is completed) and (2) that natural objects are more structurally similar to one another, compared to manmade ones, in terms of contour overlap and constituent parts. As a result, according to this cascade model, natural objects will activate more structural descriptions and there will be increased competition between category exemplars for individual identification during naming.

However, during the last decades, feedforward and cascading views on cognitive dynamics have given way to more interactive views involving recurrent processing between different brain areas (Ahissar & Hochstein, 2004; Bar, 2003; Graboi & Lisman, 2003; Lee & Mumford, 2003; O’Reilly, Wyatte, Herd, Mingus, & Jilk, 2013). For example, unlike most traditional recognition models that assume that feedforward activity causes the activation of the correct object-node, Graboi and Lisman (2003) have shown how bidirectional flow of information in reciprocally connected hierarchical cortical areas can be organized to produce recognition of objects through the detection of combinations of features, and how the serial process of attention can be integrated with the parallel recognition processes. After the early activation of a set of candidate objects based on the quickly extracted low spatial frequencies (Bar, 2003), later bottom-up flow of detailed information through a narrow window of attention then leads to the inactivation (exclusion) of candidate objects that are inconsistent with the sampled information, thereby reducing the set of possible candidate object identities. Algorithms for moving attention make use of top-down connections to compute the relative probability of each feature, given the set of still-possible objects, which will determine the subsequent location of attention. Recognition occurs after a few cycles when the serially sampled information leads to the inactivation of all but one candidate object (Graboi & Lisman, 2003). The observation that activity in object-related areas of the brain increases during the prerecognition period (Eger, Henson, Driver, & Dolan, 2007) is consistent with a decreasing competition between activated candidate representations.

## Visual complexity and basic-level categories

According to Donderi (2006), the visual complexity of a single form can be measured in two ways, either with or without reference to other forms. Panis and Wagemans (2009) suggest that this distinction is reflected in the role of two different perceptual processes during object identification. The ease of grouping image elements (segments into contours, contours into parts, parts into structural descriptions) will depend on the complexity of the visual form as such, while matching the segregated figure to memory will depend on the complexity (or similarity) of the visual form in relation to stored object representations. Because shapes with a high (or low) complexity-as-such also have a low (or high) a priori probability of occurrence in nature (Donderi, 2006), we cannot only expect that fragmented outlines of low complex shapes are a priori easier to group, but also that they will activate a larger number of candidate objects early in processing compared to high complex shapes, and that later matching and decisional processes will last longer (see also Gerlach & Marques, 2014).

The complexity of a stimulus as such (without reference to other forms) can be defined in a number of ways (the number of parts, saliency of parts, global symmetry, contour complexity, etc.). The saliency of the parts within the outline will influence whether low spatial frequencies contain diagnostic information. Low spatial frequency information or coarse global shape information is believed to be processed faster and used to activate possible candidate object representations in a top-down fashion (Bar, 2003). For objects with low part saliency (i.e., globally convex, more circle-like outlines), the low spatial frequencies contain less diagnostic basic-level shape information compared to outlines with high part saliency.

The second way in which complexity can be measured is with reference to other forms (Donderi, 2006). According to the visual crowding hypothesis, category-specific impairments in object processing in normality and in pathology can be the result of processing differences in pre-semantic stages, because natural objects are more structurally similar than artefacts (Humphreys & Forde, 2001; Humphreys et al., 1988), and therefore they can be said to have a lower complexity than artefacts (although without reference to other forms, the complexity of animals may be higher than that of artefacts; Panis, Vangeneugden, & Wagemans, 2008).

## Category-selectivity in interactive theories

To explain why *fragmented* line-drawings of natural objects are named *faster* by normal observers than manmade objects – a finding that the cascade model cannot explain – Gerlach and colleagues (Gerlach et al., 2002; Gerlach, Law, & Paulson, 2004; Gerlach, Law, & Paulson, 2006) have suggested that the structural similarity between stored exemplars of different categories affects the grouping and the matching processes that are required to access a stored object shape representation (or structural description) in a fundamentally different way. High structural similarity between stored exemplars can be advantageous for integrating local object segments and parts into whole object representations because the global and local features of these exemplars are more stable and more highly correlated than the features of exemplars from categories with low structural similarity. At the same time however, high structural similarity may be harmful for matching operations, because there will be more competition between activated integral units for object selection or covert identification (i.e., deciding that a single match has been found with stored object information in visual long-term memory).

As a result, Gerlach et al. (2002, 2004, 2006) found that, under optimal grouping conditions, i.e., with complete line-drawings and unlimited exposure, high complex objects (with low structural similarity, e.g., artefactual objects) are named faster and more accurately because there is less competition at the level where activated object representations compete for selection (a matching advantage), compared to low complex objects (with high structural similarity, e.g., natural objects). In contrast, in tasks where the demand on perceptual differentiation is not too high (e.g., for naming, not for difficult object decision tasks) and under suboptimal grouping conditions (e.g., fragmentation, limited exposure duration), low complex objects (with high structural similarity, e.g., animals) can be named faster and more accurately because: (1) under such conditions task performance tends to depend on global shape information carried by low spatial frequencies and (2) outlines and silhouettes of natural objects are better identifiable than those of artefacts, which are believed to rely more on a part-based description (Riddoch & Humphreys, 2004), while the global shape of natural objects might contain more salient features or less 2D/3D ambiguity (Lloyd-Jones & Luckhurst, 2002; Wagemans et al., 2008). As a result, early feedback information from (the current set of) activated candidate object representations can influence difficult grouping and segmentation processes in posterior IT (Gerlach et al., 2002; see also Grill-Spector & Kanwisher, 2005; Ullman, 2007), and the global shape characteristics of activated natural objects will produce a grouping advantage under suboptimal grouping conditions, which can outweigh their disadvantage during matching under optimal conditions (Gerlach et al., 2004, 2006).

## Current study

The present neuropsychological study builds on previous extensive investigations of these principles in healthy volunteers, using short presentation times and survival analysis. In one study, Panis and Wagemans (2009) recorded how many masked (re)presentations of a highly fragmented outline (with 79% of the outline deleted) were required before it was correctly recognized at the basic level (with presentation duration slowly increasing from 80 ms for the first presentation to 200 ms for the possible tenth presentation). Using discrete time survival analysis they performed a microgenetic analysis of the development over time of the effects of contour integration cues (density of fragments, proximity, and collinearity between fragments), fragment properties (their curvature), stimulus complexity-as-such (global symmetry, part saliency, and the number of parts), and memory factors (the structural similarity between stored exemplars from natural and artefactual object categories) on grouping and matching processes leading to identification.

They found that the effects of most of these manipulations on identification response occurrence changed with the passage of time. Four results are relevant for the current study. First, the effect of symmetry was maximal on the first presentation and quickly decreased over time. Second, complexity as such (independent from object category) influenced grouping negatively and matching positively (Donderi, 2006; Gerlach & Marques, 2014). Third, there was an advantage for natural objects over artefactual objects as predicted by Gerlach et al. (2004, 2006) but only for complex outlines, suggesting that top-down facilitation from activated candidates is only helpful if the low spatial frequencies contain enough diagnostic information to limit the number of activated candidate objects. Finally, straight fragments were especially beneficial for grouping the fragments of complex object outlines; an effect that was present at the first presentations. This is consistent with the proposed extrapolation cost for curvature (Singh & Fulvio, 2005), that is, straight segments convey more direction information compared to curved segments of the same length. In contrast, curved fragments were especially beneficial for (top-down) matching of simple object outlines and this effect emerged only at later presentations, i.e., for objects not identified after the first few (re)presentations. This is consistent with the idea that the details of the location and curvature of fragments is important to eliminate activated incorrect candidates (Biederman, 1987; Graboi & Lisman, 2003).

In the current study, we perform a similar microgenetic analysis on the naming performance of neurologically healthy individuals and patient GA, a single case showing a category-selective impairment for natural objects due to a HSE infection, as a way to investigate what GA’s performance patterns can tell us about the origin of category-specific naming deficits. Specifically, if GA’s naming impairment is the result of his loss of high-level structural descriptions, then the naming advantage for natural objects with fragmented object outlines found with healthy individuals should be absent, because the activated candidates cannot top-down constrain the recurrent grouping of parts into a structural description (which would benefit natural object categories more than artefactual ones because of their higher structural similarity).

To maximize the performance and motivation of our patient, we use a dynamic build-up paradigm with long presentation times and with fragments increasing in length in 10 steps, starting with 10% of the contour for the first presentation and ending with 100% for the tenth presentation. We test the effects of complexity as such (using a measure of symmetry and a measure of outline complexity, i.e., homogeneity), category (manmade versus natural), and fragment curvature (straight vs. curved). Although Torfs, Panis, and Wagemans (2010) showed that the effects of fragment curvature were absent for healthy observers in a dynamic build-up paradigm with long presentation times (while present in Panis and Wagemans [2009] who used very short masked presentation times), we still include fragment curvature to study whether it influences the performance of patient GA.

Just as Panis and Wagemans (2009), we use survival analysis (Allison, 2010; Panis & Hermens, 2014; Panis & Schmidt, 2016; Singer & Willett, 1993, 2003) to investigate how the category-specific impairment of patient GA depends on: (1) the time required to identify a fragmented object outline at the basic level and (2) local (fragment curvature) and global (symmetry and shape complexity) aspects of the fragmented object outlines, compared to normal controls. Survival analysis is the standard set of statistical techniques used to analyse time-to-event data such as recognition latency data. The reason is that the mean and standard deviation are inappropriate statistics when analysing time-to-event data because it is always possible that you do not measure a response in a trial (more generally: the data collection period). Indeed, in 43% of the trials, GA could not identify the object after the final (tenth) presentation. This missing-data problem is known as “right-censoring”. Ignoring such right-censored trials will result in a sample mean that seriously underestimates the “true” mean because right-censored trials do contain the information that the response must be larger than the data collection period. Survival analysis deals evenhandedly with observed and censored response times, and allows us to study whether and how the effect of a manipulation on instantaneous response occurrence *changes* with the passage (i.e., increase) of waiting time (Singer & Willett, 2003).

We test the following dynamic predictions about naming performance in our build-up paradigm with fragmented object outlines. First, for normal controls we expect better performance with natural objects, but only for the first few presentations when highly fragmented outlines are shown, because structural similarity is only beneficial for grouping the fragments of structurally similar (i.e., natural) objects through feedback from early activated candidate structural representations (Gerlach, 2009). Second, only during the latest presentations when grouping is not an issue anymore we expect better performance for manmade compared to natural objects for normal controls, as observed by Humphreys et al. (1988). Third, for patient GA, who has impairments in structural and semantic knowledge, the early advantage for natural objects as predicted for healthy volunteers should be strongly reduced or absent, because of less efficient top-down guidance from stored structural descriptions during grouping. Fourth, during the latest presentations we also expect an advantage for manmade objects for patient GA.

## Methods

### Participants

**Figure 1.**
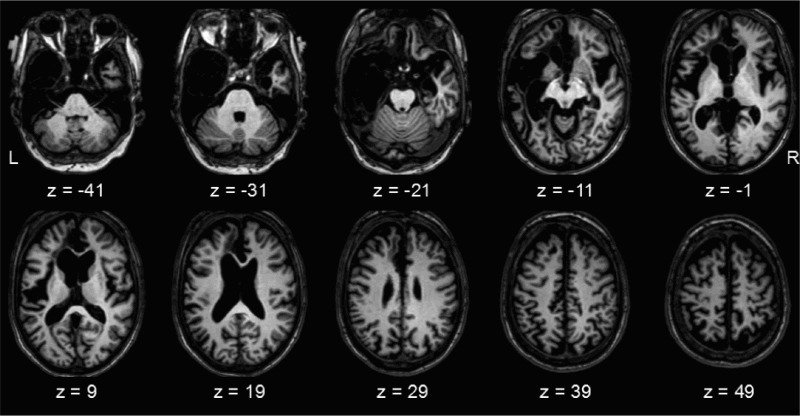
Anatomy of patient GA’s brain on a T1-weighted magnetic resonance image, normalized to MNI space using the clinical toolbox implemented in SPM8 (www.fil.ion.ucl.ac.uk/spm).

Patient GA is a 57-year-old male (DOB 23 May 1954) and former professional musician. In 1990 he suffered a HSE infection that resulted in bilateral damage to the middle and anterior temporal lobe regions which extended forward into the frontal lobes (particularly on the left). The anatomy of GA’s brain is depicted in Figure 1. He has a deficit in object identification, which is most pronounced for natural objects. There is no evidence of a low-level perceptual deficit (Humphreys & Riddoch, 2003).

Based on data collected between 2000 and 2002, Humphreys and Riddoch (2003) showed that GA: (1) was somewhat impaired at naming pictures from manmade objects (vehicles, furniture, tools, musical instruments), strongly impaired at naming pictures from natural categories (animals, fruit, vegetable), but not impaired for body parts, (2) performed below the control level when tested for access to stored structural and semantic knowledge, and (3) had difficulties in discriminating between perceptually similar living things.

Seven control participants with no history of neurological disease were also included in the study (one female, average age = 67 years, *SD* = 11 years). All participants had normal or corrected-to-normal vision, were unfamiliar with the stimuli, and naive regarding the purpose of the study.

All participants signed an informed consent and the study was approved by the local ethics committee.

### Neuropsychological profile

The cognitive profile of patient GA was derived using the Birmingham Cognitive Screen (BCoS; Humphreys, Bickerton, Samson, & Riddoch, 2012), which is an extensive cognitive screen designed to detect cognitive impairments in different domains, including memory, language, attention and executive functioning, praxis, and number processing (Table 1).

**Table 1.** Neuropsychological profile of GA on the Birmingham Cognitive Screen (BCoS).

DOMAIN	Subdomain	Function	Range	GA
MEMORY	Orientation	Personal	0–8	6
		Time & Space	0–6	6
	Long term	Recall	0–15	0
		Recognition	0–15	5
	Short term	Recall	0–15	1.5
		Recognition	0–15	5
	Episodic	Task recognition	0–10	3
LANGUAGE	Spoken	Picture naming	0–14	2
		Sentence construction	0–8	6
	Comprehension	Comprehension	1–3	3
	Writing	Sentence reading	0–42	39
		Non-word reading	0–6	5
		Writing	0–5	2
ATTENTION	Spatial neglect	Overall	0–50	48
		Page Asymmetry	0–20	0
		Object Asymmetry	0–50	0
	Spatial extinction	Visual - Left space	0–8	8
		Visual - Right space	0–8	8
		Tactile - Left space	0–8	8
		Tactile - Right Space	0–8	8
	Executive function	Rule accuracy	0–18	16
		Total rules	0–3	3
	Auditory attention	Total accuracy	0–54	44
		Working memory	0–3	2
		Sustained attention	0–18	7
PRAXIS	Action	Object use	0–12	9
		Gesture production	0–12	11
		Gesture recognition	0–6	4
		Imitation	0–12	12
		Figure copy	0–47	46
NUMBER		Reading	0–9	8
		Writing	0–5	4
		Calculation	0–4	2

Note: Underlined values indicate worse performance for patient GA compared to the norms (Humphreys et al., 2012).

### Stimuli

The stimulus set consisted of one intact outline and 18 fragmented versions for each of 100 line-drawings of objects (a subset of the stimuli used by Torfs et al., 2010). Two types of contour deletion or fragmentation were used (Panis, De Winter, Vandekerckhove, & Wagemans, 2008). Fragments were either placed around salient points (SP; usually near extrema on the contour, see De Winter & Wagemans, 2008) or around midpoints between two salient points (MP). Because SPs typically have large curvature values, small SP-fragments are usually strongly curved. Small MP-fragments, on the other hand, are relatively straight, and convey more direction information compared to curved fragments of the same length (Singh & Fulvio, 2005). A more detailed description of outline construction and fragmentation can be found in Wagemans et al. (2008; see also De Winter & Wagemans, 2004; Wagemans, Notebaert, & Boucart, 1998).

For this study, we selected a subset of 100 objects (Table S1 in Supplemental data) with identification rates in healthy volunteers of at least 70% in contour version, and at least 70% in silhouette version (Wagemans et al., 2008). The identification rate of the selected objects was at least 90% in the build-up study by Torfs et al. (2010). Thus, we can expect that controls will identify most objects before the end of the presentation sequence. A total of 51 of the stimuli were manmade (including vehicles, furniture, clothing) and 49 natural (including animals, fruits, vegetables). These two groups did not differ in average complexity of the stimuli, nor in mean thresholds of recognition in fragmented version (see Table 2 for *t*-tests).

**Table 2.** Mean object image statistics for the selected set of 100 objects.

		Manmade	Natural	*t*	*df*	*p*
Mean % identification	Contour	96.53	97.48	−0.968	98	0.336
	Silhouette	97.04	98.09	−1.099	98	0.274
	Fragmented	98.83	99.13	−0.768	98	0.444
Proportion symmetrical objects		0.51	0.31	2.095	98	0.039
Average complexity (homogeneity)		26.35	21.45	0.634	98	0.527
Mean threshold of identification in MP version		4.44	3.86	1.732	98	0.086
Mean threshold of identification in SP version		4.62	4.02	1.788	98	0.077

Notes: This table includes some mean image statistics of the final set of 100 stimuli: *t*-statistics, and their *p-*values. (Identification measures: De Winter & Wagemans, 2004; Torfs et al., 2010; complexity measure: Panis & Wagemans, 2009). The identification measures are based on large-scale studies, and reflect correct naming by participants without a history of brain damage.

**Figure 2.**
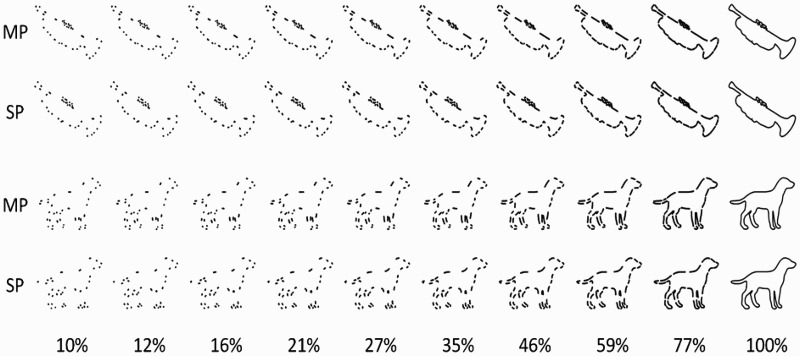
Examples of fragmented outlines (trumpet and dog) in two fragmentation types (MP and SP). Note that the stimuli in the experiment were presented in white on a black background.

As in Torfs et al. (2010), we calculated the percentage contour shown at each level x (x = 1, 2, … 10) as 100 times alpha^(10–x)^ (Snodgrass & Corwin, 1988) instead of a linear increase of percentage contour. For the build-up process to start at 10%, alpha was set to .77. The function resulted in the following percentages: 10, 12, 16, 21, 27, 35, 46, 59, 77, and 100 (the intact outline). Compared to a linear function, less perceptual information was added during early levels (and more later) to slow down the recognition process. Starting from the relevant set of target points (MPs or SPs), the requested percentage contour shown at each level was approximated by letting the fragments grow until the requested percentage was reached while taking into account the distances along the contour between the target point and both of its neighbouring points. As a result, the same number of equally evenly distributed fragments was presented for each fragmented version of an object outline. Examples of fragmented object outlines can be found in Figure 2.

### Apparatus

The stimuli were presented centrally on a 17-inch CRT display with a refresh rate of 60 Hz and at a viewing distance of approximately 60 cm. The display resolution was set to 1024 by 768 pixels. Stimuli were all contained within a box of 640 by 480 pixels, resulting in a viewing angle of about 16 by 12 degrees. E-prime (www.pstnet.com) was used to deliver presentation times. All participants were tested individually.

### Procedure

**Figure 3.**
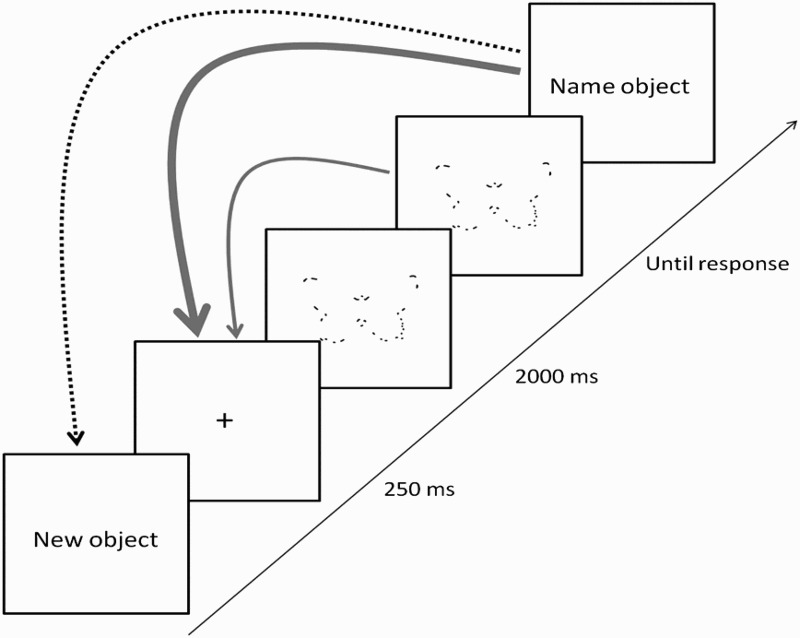
The dynamic build-up paradigm. Trials started with a 250 ms fixation cross, and were followed by a fragmented object outline presented for 2 s. When a negative response was registered, “I need more information” or “I don’t know what it is”, the build-up of the outline continued (thin full arrow). When a positive response was registered (i.e., object name) but the answer was incorrect, the build-up of the outline continued (thick full arrow). When a correct response was given, the build-up of the current object outline was aborted and the build-up of the next object started (dashed arrow).

We used a similar procedure to Torfs et al. (2010). A schematic presentation of the procedure is shown in Figure 3. Each trial consisted of one or more presentations of a fragmented version of the same object. Trials were self-paced and started with a fixation cross for 250 ms, followed by an object outline at fragmentation level 1 (10% of the contour) in one of both fragmentation conditions (MP or SP). The fragmented outline was presented for at least 2000 ms, that is, until the participant indicated that (s)he recognized the object or needed more contour information. We used a relatively long presentation time of 2 s because patient GA was easily distracted, reflecting his executive problems. If a participant indicated verbally that (s)he had recognized the object, the stimulus presentation was interrupted by the experimenter (i.e., the stimulus disappeared from the screen) and the participant was asked to name the object aloud. The experimenter evaluated the response on-line (see below). When the response was scored as correct, a new object outline (fragmentation level 1) was shown in the next trial. When the response was incorrect, the build-up of the current outline continued (possibly showing 12, 16, 21, 27, 35, 46, 59, 77, and 100% of the contour). Participants were given feedback about the accuracy of their answer and they were informed about the presence of a new object before its first presentation.

All participants saw each object twice (once in each fragmentation type) resulting in a total number of 200 trials per subject. For each combination of object and participant, we recorded the lowest presentation number (1–10) that resulted in correct naming.

The presentation order of the objects was randomized for each participant separately, taking into account that an object could not appear (i.e., in the other fragmentation type) on two subsequent trials. Patient GA needed six sessions of 1 h to complete the experiment. Control participants completed the experiment in two sessions, each lasting 1 h at most. At the beginning of each session, there were some practice trials with a separate set of four objects (same set for all participants).

### Scoring

We used the same scoring rules as Panis and Wagemans (2009). A response was counted as correct when the same name was given as the one listed by Snodgrass and Vanderwart (1980), or a synonym or dialect name that clearly indicated the same concept. We also approved names referring to closely related objects if these were not visually distinguishable in the object outlines (e.g., “mouth” for “lips” or “rat” for “mouse”). However, we did not allow related names that referred to a different basic-level and visually distinguishable category (e.g., “shoe” and “boot” or “chicken” and “bird”). Scoring was done manually and on-line by the experimenter using the scoring key based on a naming database from previous studies (e.g., De Winter & Wagemans, 2004; Torfs et al., 2010; Wagemans et al., 2008).

### Analysis

To investigate whether and when shape complexity, object category, and local contour information (MP vs. SP) influence correct (basic level) identification of natural and manmade objects in patient GA and healthy volunteers, we used discrete time survival analysis (Panis & Schmidt, 2016; Panis & Wagemans, 2009). We will give only a short description of the main features of this analysis technique here, and refer the reader for more elaborate explanations to Singer and Willett (1993, 2003) and Allison (2010).

The target event in this study is a correct identification response, and the response time is the number of presentations between the first presentation and the occurrence of the target event. This gives rise to interval-censored data: we know only that correct identification occurred somewhere between the onsets of two subsequent presentations (or never in case of right-censored observations).

Ideally, the time between stimulus onsets should be constant in order to interpret the effect of time across conditions (in our case, between controls and GA). However, this was not feasible for patient GA because he could be distracted at unpredictable times during the build-up. Therefore, we measured the time needed to identify an object in terms of the number of presentations shown while being aware that there is no perfect correlation between that number and the actual time in ms taken to identify the object (see Panis and Schmidt [2016] for an application of survival analysis to response time data).

Because we are dealing with waiting times, the distribution of event occurrence is best summarized by two statistics: the hazard and survivor functions. The discrete-time hazard probability *h*(*t*) is the conditional probability that the stimulus will be correctly identified after presentation *t*, given it has not been identified after earlier presentations, or *h*(*t*)* = P*(*T = t | T ≥ t*). The survivor function cumulates the risks of event non-occurrence, and gives the probability that the stimulus will “survive” (i.e., not be identified) presentation *t*, or *S*(*t*)* *=* P*(*T *>* t*)* = *[*1–h*(*t*)].[*1–h*(*t–1*)].[*1–h*(*t–2*)]* * … * *[*1–h*(*1*)]. Because the hazard function is bounded between 0 and 1, we need to apply a transformation before generalized linear models (GLM) for repeated measurements can be fitted to the data. We applied the nonlinear, asymmetric complementary log-log link function (cloglog[*h*(*t*)] = ln(−ln(1–[*h*(*t*)]))), because this transformation is most attractive when discrete time methods are used while the underlying metric of time is truly continuous (Singer & Willett, 2003).

To fit discrete time hazard models to the data using logistic regression, the subject-by-trial oriented dataset has to be expanded to a subject-by-trial-by-time bin (i.e., presentation number) oriented dataset, where an EVENT variable indicates event occurrence (1/0) for each presentation period that is at risk of event occurrence, and a TIME variable indicates the rank of each presentation (minus 1 when centring TIME on presentation 1). The basic discrete time hazard model can be written as follows: cloglog [*h*(*t*)] = [*α*_0_ONE + *α*_1_(TIME – 1) + *α*_2_(TIME – 1)^2^ + *α*_3_(TIME – 1)^3^] + [*β*_1_ *X*_1_ + *β*_2_ *X*_2_ + … + *β_P_X_P_*]. The first set of terms within brackets, the alpha parameters multiplied by their polynomial specifications of (centred) time, represents the *baseline* cloglog hazard function (i.e., when all predictors *X*_i_ take on a value of zero). The second set of terms (the beta parameters) represents the (vertical) shift in the baseline hazard when their respective dichotomous predictors take on a value of 1. To interpret the effects of the dichotomous predictors, the parameter estimates are anti-logged, resulting in a hazard ratio (HR).

The dichotomous predictors include *patient* (1 for GA, 0 for controls), *object category* (1 for manmade, 0 for natural objects), global *symmetry* (1 for present, 0 for absent), *complexity* of the object outline (1 = complex, 0 = simple), and *fragment type* (MP = 1, SP = 0). The full model includes all the main effects, all two-way interactions between the variables, the linear, quadratic, and cubic main effect of *time* (i.e., presentation number, PT), and the linear and quadratic interaction effects between time and the other main and interaction effects. Because all objects were presented twice (once in each fragmentation type), the effect *Trial Repetition* (0 for first trial, and 1 for second trial of the same object) was included in the model to statistically control for it. We used a backward model selection procedure, and applied the hierarchical principle. The SURVEYLOGISTIC procedure of SAS 9.3 was used to fit the hazard models to the data, with the complementary log-log transformation as a link function.

## Results

**Figure 4.**
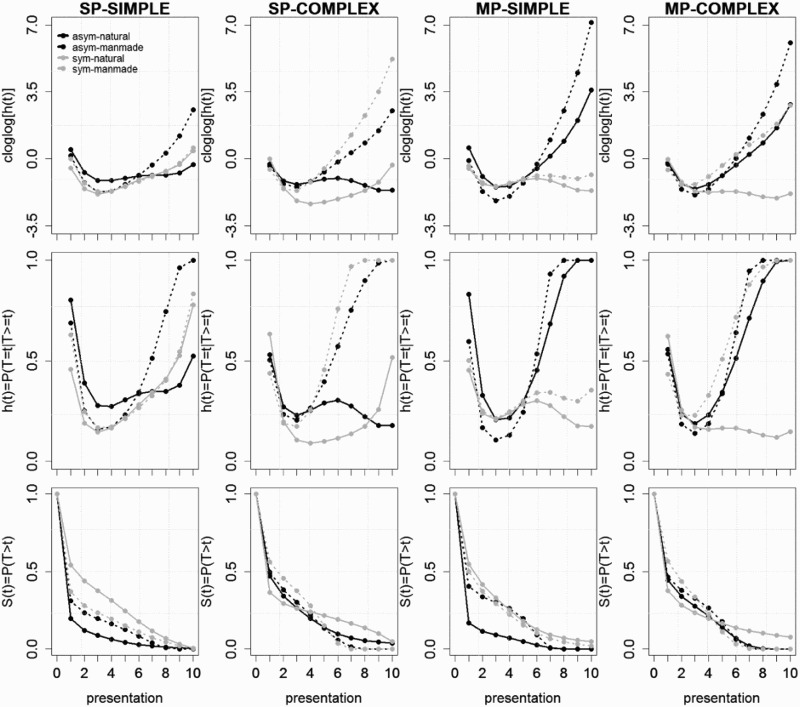
Model-based functions. Predicted cloglog[*h*(*t*)], *h*(*t*), and *S*(*t*) functions for controls during the trials in which the object is presented for the first time. asym = asymmetry, sym = symmetry.

**Figure 5.**
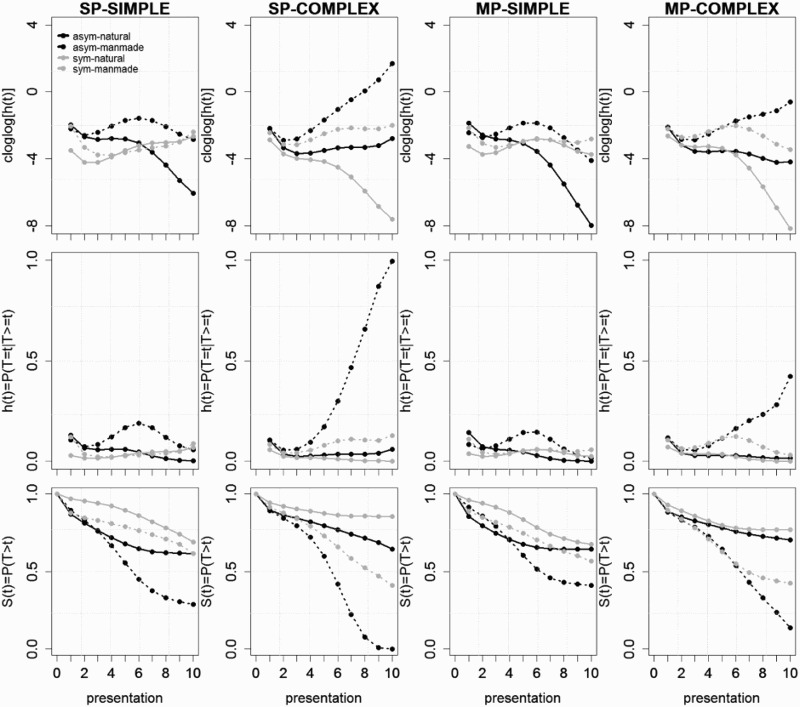
Model-based functions. Predicted cloglog[*h*(*t*)], *h*(*t*), and *S*(*t*) functions for GA during the trials in which the object is presented for the first time. asym = asymmetry, sym = symmetry.

The overall percentage of correct identification responses was 57% for GA and at ceiling (99%) for control subjects. To test whether the main and interaction effects-of-interest were significant across participants and change across time or presentation number, we fitted discrete-time hazard models to the aggregated data. The predicted *cloglog*[*h*(*t*)] functions from the selected model for the normal controls are shown in Figure 4, as well as the predicted *h*(*t*) functions, which were obtained by applying the inverse of the *cloglog* link function. Specifically, an increase in cloglogs by an additive constant *a* corresponds to a multiplicative increase in hazards (or *hazard ratio, HR*) by a factor of exp(*a*). The corresponding survivor functions *S*(*t*) = *P*(*T* > *t*) are shown in row 3 in Figure 4. The predicted functions for GA are shown in Figure 5. The parameter estimates (PEs) and test statistics are shown in Table 3. During model selection, TIME was centred on the first presentation so that any numerical values of main effects and interactions not explicitly involving TIME refer to the first presentation of an object within a trial. After initial model selection, we refitted the selected model four times with TIME recentred on presentations 3, 5, 7, and 9 to see explicitly what values the parameter estimates take on after these presentation numbers, and whether they represent a significant effect or not (see columns 7–14 in Table 3).

**Table 3.** The final discrete time hazard model.

			Presentation 1			Presentation 3		Presentation 5		Presentation 7		Presentation 9	
			(10% contour)			(16% contour)		(27% contour)		(46% contour)		(77% contour)	
No.	Effect	PE	Standard Error	Z	Pr > |Z|	PE	Pr > |Z|	PE	Pr > |Z|	PE	Pr > |Z|	PE	Pr > |Z|
1	Intercept	0.4812	0.1398	11.8569	0.0006	−1.1279	<.0001	−1.0087	<.0001	−0.8516	0.0512	−0.7417	0.5282
2	PT	−1.7191	0.1738	97.7837	<.0001								
3	PT^2^	0.6114	0.0506	145.981	<.0001								
4	PT^3^	−0.0854	0.0123	48.4214	<.0001								
5	PT^4^	0.00418	0.00076	30.2603	<.0001								
6	**Trial repetition**	**0.5968**	0.0963	38.3929	<.0001	**0.5968**	<.0001	**0.5968**	<.0001	**0.5968**	<.0001	**0.5968**	<.0001
7	**Manmade**	−0.3262	0.1871	3.0397	0.0813	−**0.6101**	0.0156	−0.3264	0.2329	0.5248	0.2945	1.9435	0.0734
8	Manmade*PT	−0.2838	0.1522	3.4762	0.0623								
9	Manmade*PT^2^	0.0709	0.0325	4.7682	0.029								
10	**Symmetry**	−**0.9747**	0.2245	18.8576	<.0001	−**0.7251**	0.019	−**0.4044**	0.0308	−0.0127	0.9773	0.45	0.7221
11	Symmetry*PT	0.107	0.1802	0.3528	0.5525								
12	Symmetry*PT^2^	0.00888	0.0421	0.0444	0.833								
13	Manmade*Symmetry	**0.8124**	0.2637	9.4882	0.0021	**0.7615**	0.0165	0.2944	0.1873	−0.5891	0.1687	−1.8889	0.0903
14	Manmade*Symmetry*PT	0.0787	0.1465	0.2883	0.5913								
15	Manmade*Symmetry*PT^2^	−0.052	0.0352	2.1856	0.1393								
16	**Complex**	−**0.7629**	0.1613	22.368	<.0001	−0.22	0.3533	−0.0593	0.8282	−0.2807	0.4455	−0.8842	0.2621
17	Complex*PT	0.367	0.1719	4.5583	0.0328								
18	Complex*PT^2^	−0.0478	0.0293	2.6635	0.1027								
19	Manmade*Complex	**0.2497**	0.1094	5.2081	0.0225	**0.4798**	<.0001	**0.7098**	0.0039	**0.9399**	0.0201	**1.17**	0.0382
20	Manmade*Complex*PT	0.115	0.0809	2.0228	0.155								
21	Symmetry*Complex	**1.2609**	0.1714	54.1072	<.0001	−0.1223	0.7041	−**0.7979**	0.0116	−**0.7659**	0.0088	−0.0264	0.975
22	Symmetry*Complex*PT	−0.8685	0.2245	14.9702	0.0001								
23	Symmetry*Complex*PT^2^	0.0884	0.0389	5.1704	0.023								
24	Manmade*Symmetry*Complex	−**1.292**	0.2304	31.4403	<.0001	−0.0973	0.6591	**1.0975**	0.0008	**2.2923**	<.0001	**3.4871**	<.0001
25	Manmade*Symmetry*Complex*PT	0.5974	0.0888	45.2136	<.0001								
26	**MP**	0.0926	0.2451	0.1428	0.7055	−0.3391	0.1621	−0.04	0.801	**0.9901**	0.0345	**2.751**	0.0231
27	MP*PT	−0.3986	0.1668	5.7112	0.0169								
28	MP*PT^2^	0.0914	0.0383	5.6875	0.0171								
29	Manmade*MP	−0.3464	0.3001	1.3323	0.2484	−0.1232	0.632	0.1001	0.6737	0.3234	0.1913	0.5466	0.0537
30	Manmade*MP*PT	0.1116	0.0423	6.9592	0.0083								
31	Symmetry*MP	−0.1032	0.1469	0.4939	0.4822	**0.74**	0.0002	**0.3549**	0.0447	−**1.2584**	0.0052	−**4.1**	0.0001
32	Symmetry*MP*PT	0.7287	0.147	24.5795	<.0001								
33	Symmetry*MP*PT^2^	−0.1535	0.0324	22.4171	<.0001								
34	Complex*MP	−0.0189	0.1875	0.0102	0.9197	0.109	0.4256	0.2369	0.103	0.3648	0.0762	0.4927	0.0861
35	Complex*MP*PT	0.0639	0.0485	1.7371	0.1875								
36	Manmade*Complex*MP	**0.3601**	0.1692	4.5279	0.0333	−0.07	0.7319	−0.5001	0.0738	−**0.9301**	0.0124	−**1.3602**	0.0039
37	Manmade*Complex*MP*PT	−0.215	0.0542	15.7596	<.0001								
38	**Patient**	−**2.4522**	0.0966	644.4005	<.0001	−**1.7005**	<.0001	−**1.7967**	<.0001	−**2.7408**	<.0001	−**4.5328**	0.0002
39	Patient*PT	0.5878	0.191	9.4702	0.0021								
40	Patient*PT^2^	−0.106	0.0423	6.2656	0.0123								
41	Patient*Manmade	0.0988	0.1125	0.77	0.3802	**1.0101**	<.0001	**1.4338**	<.0001	**1.3697**	<.0001	0.8178	0.3841
42	Patient*Manmade*PT	0.5776	0.1282	20.3075	<.0001								
43	Patient*Manmade*PT^2^	−0.061	0.031	3.8586	0.0495								
44	Patient*Symmetry	−**0.5621**	0.1481	14.4003	0.0001	−**0.6621**	0.0185	−0.2908	0.0699	0.5518	0.2039	1.8658	0.1447
45	Patient*Symmetry*PT	−0.1678	0.1878	0.7992	0.3713								
46	Patient*Symmetry*PT^2^	0.0589	0.044	1.7948	0.1803								
47	Patient*Manmade*Symmetry	**0.8693**	0.2014	18.6388	<.0001	−**0.7167**	0.0099	−**1.5404**	<.0001	−**1.6017**	<.0001	−0.9007	0.372
48	Patient*Manmade*Symmetry*PT	−0.9836	0.1265	60.4669	<.0001								
49	Patient*Manmade*Symmetry*PT^2^	0.0953	0.0317	9.0246	0.0027								
50	Patient*Complex	**0.5244**	0.0871	36.2648	<.0001	−**0.6399**	0.0029	−**0.6238**	0.0093	**0.5728**	0.044	**2.9498**	<.0001
51	Patient*Complex*PT	−0.8772	0.164	28.6032	<.0001								
52	Patient*Complex*PT^2^	0.1476	0.0288	26.2467	<.0001								
53	Patient*Symmetry*Complex	−**0.3681**	0.109	11.4144	0.0007	**1.2133**	<.0001	**0.8155**	<.0001	−**1.5614**	<.0001	−**5.9176**	<.0001
54	Patient*Symmetry*Complex*PT	1.2855	0.2309	30.9987	<.0001								
55	Patient*Symmetry*Complex*PT^2^	−0.2474	0.0435	32.3873	<.0001								
56	Patient*MP	0.0131	0.0836	0.0246	0.8754	**0.3665**	0.0237	−**0.2272**	0.0008	−**1.7681**	0.0004	−**4.2562**	0.001
57	Patient*MP*PT	0.4135	0.162	6.5149	0.0107								
58	Patient*MP*PT^2^	−0.1184	0.0398	8.8388	0.0029								
59	Patient*Symmetry*MP	**0.2481**	0.1216	4.1629	0.0413	−0.1751	0.3393	**0.4794**	0.0068	**2.2117**	<.0001	**5.0216**	<.0001
60	Patient*Symmetry*MP*PT	−0.481	0.1711	7.904	0.0049								
61	Patient*Symmetry*MP*PT^2^	0.1347	0.0368	13.4281	0.0002								

Notes: This includes the 61 parameter estimates (PE) of the reduced model, their standard errors, test statistics, and *p*-values (columns 3–6). Significant PE (*p* < .05) not involving TIME are indicated in bold. The other columns show how PE and *p*-values change when TIME (i.e., presentation number, PT) is recentred on later presentations within a trial.

### Control participants: SP-fragmented simple outlines

The first five parameter estimates in column 3 of Table 3 model the shape of the *cloglog*[*h*(*t*)] function for the baseline condition: SP-fragmented, simple, asymmetrical, natural object outlines identified by controls during the trials in which the object is presented for the first time (Figure 4, row 1, column 1, full black line). Because of the centring of the variable TIME (i.e., presentation number, PT), the intercept of our regression model refers to the model estimate after presentation 1. The predicted *cloglog*[*h*(*1*)] value is 0.4812, when the effects Trial repetition, Symmetry, Manmade, Complex, MP, and Patient are set to zero. Converting back from cloglogs to hazards, *h*(*1*) = .80 (= 1 – exp[−exp(0.4812)]; Figure 4, row 2, column 1, presentation 1). Parameters 2–5 show a significant linear, quadratic, cubic, and quartic effect of TIME on this intercept estimate, such that the predicted hazard of correct identification in the baseline condition first decreases and then steadily increases over presentations: *h*(*1*) = .80, *h*(*3*) = .28, *h*(*5*) = .31, *h*(*7*) = .35, and *h*(*9*) = .38. So, for example, given that correct identification did not occur after presentations 1 and 2, then the hazard probability of correct identification after presentation 3 or *h*(3) = *P*(*T* = 3 | *T* ≥ 3) equals .28. The conditional probability or hazard of correct identification occurrence in this baseline condition is thus already relatively high after the first presentation (10% contour shown), drops for the following two presentations (giving no correct identification on previous presentations), and then slowly increases from the fourth presentation (21% contour shown) onwards (giving no correct identification on previous presentations). Note that the survival probability *S*(5) = *P*(*T* > 5) equals .05 in the baseline condition (Figure 4, row 3, column 1, black line). Thus, only 5% of the objects in the baseline condition have not been identified correctly after the fifth presentation. Given this high and fast performance of controls, we only focus on the first five presentations for control participants, because once *S*(*t*) drops below about .1 the estimates of *h*(*t*) become unreliable.

The significant effect of Trial Repetition is time-invariant (parameter 6 in Table 3, 0.5968 in cloglog hazard units, *p* < .0001). Thus, compared to the first trial in which a unique object is shown, the hazard of correct identification for the second trial of the same object (but with different fragment type) is estimated to be 1.82 (= e^.5968^) times higher after each presentati4on.

Now, what happens to the shape of the baseline cloglog[*h*(*t*)] function when we change to manmade (asymmetrical, simple SP-fragmented) object outlines? Parameters 7–9 show a marginally significant main effect of Manmade after presentation 1 (parameter 7, PE = −0.3262, *p* = .0813), and Manmade interacts with TIME in a significant quadratic fashion, changing from (significantly) negative to (non-significantly) positive over time. Compared to the *cloglog*[*h*(*t*)] estimates in the baseline condition (Figure 4, row 1, column 1, black line), showing an SP-fragmented simple asymmetrical *manmade* outline decreases the estimated *cloglog*[*h*(*t*)] by .3262 units after presentation 1, which corresponds to an decrease in response hazard by a factor of 0.72 (*HR*(*1*) = exp[−.3262] = 0.72). Similarly, *HR*(*3*) = 0.54, and *HR*(*5*) = 0.72 (Figure 4, row 2, column 1, full vs. dotted black lines). For SP-fragmented simple *asymmetrical* outlines we thus find an advantage for natural objects, especially after presentations 2–4, that is, for outlines not identified correctly after the initial presentation(s). The corresponding survivor function is thus lower for natural compared to manmade (simple, asymmetric, SP) outlines.

What happens to the shape of the baseline cloglog[*h*(*t*)] function when we change to symmetrical (natural, simple SP-fragmented) object outlines? Parameters 10–12 show a significant main effect of Symmetry after the first presentation (parameter 10, PE = −0.9747 in cloglog units, *p* < .001), and interactions involving TIME. Compared to the reference condition, symmetry decreases the hazard of correct identification of SP-fragmented, simple, natural outlines by a factor of *HR*(*1*) = 0.38, *HR*(*3*) = 0.48, and *HR*(*5*) = 0.67 (Figure 4, row 2, column 1, black full vs. grey full lines).

What happens to the shape of the baseline cloglog[*h*(*t*)] function when we change to manmade *and* symmetrical (simple, SP-fragmented) object outlines? Parameters 13–15 show an additional significant interaction effect between Manmade and Symmetry after the first three presentations (parameter 10, PEs = 0.8124 and 0.7615 in cloglog units, *ps* = .0021 and .0165, respectively). As can be seen in Figure 4 (row 2, column 1, grey lines), the hazard of correct identification for SP-fragmented simple *symmetrical* outlines is higher for manmade than natural categories after presentations 1 and 2.

For simple (SP-fragmented) outlines we thus observe a significant advantage for natural objects when the outline is asymmetrical, especially for the first four presentations, consistent with the first prediction. When the outline is simple and symmetrical, a disadvantage for natural objects is observed especially after the first presentation. Furthermore, the negative effect of symmetry for natural (simple SP-fragmented) outlines is absent for manmade (simple SP-fragmented) outlines. This suggests that only natural objects benefit (early) from asymmetry, consistent with the idea that top-down facilitation of grouping requires global diagnostic features which are more likely to be present in asymmetrical than in symmetrical fragmented outlines (Panis & Wagemans, 2009; see also Discussion).

### Patient GA: SP-fragmented simple outlines

What happens to the shape of the baseline cloglog[*h*(*t*)] function when patient GA performs the naming task? Parameters 38–40 show a strong significant main effect of Patient after presentation 1 (parameter 38, PE = −2.4522, *p* < .0001), and this effect interacts with TIME in a significant linear and quadratic fashion. Compared to the *cloglog*[*h*(*t*)] estimates in the baseline condition (Figure 4, row 1, column 1, black line), changing from controls to GA decreases the estimated *cloglog*[*h*(*t*)] by 2.4522 units after presentation 1, which corresponds to a decrease in correct identification hazard by a factor of *HR*(*1*) = 0.09. Similarly, *HR*(*3*) = 0.18, *HR*(*5*) = 0.17, *HR*(7) = 0.06, and *HR*(9) = 0.01. Thus, GA’s performance after each presentation was significantly lower than that of control participants (Figures 4 vs. 5, row 2, column 1, full black line).

Parameters 41–43 show a significant interaction effect between Patient and Manmade after presentations 3 (parameter 41, PE = 1.0101, *p* < .0001), 5 (PE = 1.4338, *p* < .0001), and 7 (PE = 1.3697, *p* < .0001). As can be seen in Figure 5 (row 2, column 1, full vs. dotted black lines), GA shows an advantage for manmade over natural objects for SP-fragmented simple *asymmetrical* outlines starting with presentation 3 (in contrast to controls who show an early advantage for natural categories).

Parameters 44–46 show a significant interaction effect between Patient and Symmetry after presentations 1 (parameter 44, PE = −0.5621, *p* = .0001) and 2 (PE = −0.6621, *p* = .0185). Similar to controls, GA shows an (even larger) early disadvantage for symmetrical compared to asymmetrical simple outlines (but again only for natural objects; see the following effect). Parameters 47–49 show a significant interaction effect between Patient, Manmade, and Symmetry that is positive after presentation 1 (parameter 47, PE = 0.8693, *p* < .0001) and negative after presentations 3–7. As can be seen in Figure 5 (row 2, column 1), for SP-fragmented simple *symmetrical* outlines GA shows an advantage for manmade over natural objects after presentation 1, similar to controls. In contrast to controls, there is no early advantage for natural over manmade objects for SP-fragmented simple *asymmetrical* outlines, consistent with prediction 3.

### Control participants: SP-fragmented complex outlines

In the following, we will switch to a more qualitative description of the effects. Note that from here on, all additional regression parameters have to be interpreted relative to the baseline condition: for instance, there will be an additional main effect of Complexity (a dummy variable coded 0 when the outline shape is simple and 1 when it is complex). This variable may form interactions will all the effects described so far (Manmade, Symmetry, TIME, TIME^2^).

Parameters 16–18 show a significant main effect of Complexity (parameter 16, PE = −0.7629, *p* < .001) after the first presentation. Compared to the baseline condition (SP-fragmented, simple, natural, asymmetrical), changing to complex outlines decreases the hazard of correct identification occurrence with a factor of HR(1) = 0.47 (Figure 4, row 2, column 1 versus 2, black lines). This is consistent with a problem in grouping the fragments, especially during the first presentation.

Parameters 19–25 in Table 3 show: (1) a positive interaction effect between Complex and Manmade that increases in size over time, (2) an interaction effect between Complex and Symmetry that changes over time from positive to negative, and (3) an interaction effect between Complex, Manmade, and Symmetry that is negative after the first presentation and positive after the fifth and later presentations.

As can be seen in Figure 4 (row 2, columns 1 vs. 2) the result is: (1) for complex SP-fragmented *symmetrical objects* (grey lines in Figure 4, column 2, row 2), there is an advantage for natural objects after the first presentation, which reverses to an advantage for manmade categories after the third and later presentations, and (2) for complex SP-fragmented *asymmetrical* objects (black lines), there is mainly an advantage for manmade objects after the fifth and later presentations. These observations are consistent with prediction 2. Again, the effect of symmetry is present mainly for natural objects.

### Patient GA: SP-fragmented complex outlines

Parameters 50–52 show a significant interaction effect between Patient and Complex that changes over time from positive to negative to positive. Parameters 53–55 show a significant interaction effect between Patient, Symmetry, and Complex that changes over time from negative to positive to negative. As can be seen in Figure 5 (row 2, column 2), starting around the third presentation GA shows an advantage for manmade over natural objects for both asymmetrical and (to a lesser extent) symmetrical SP-fragmented complex outlines, consistent with prediction 4.

### Control participants: MP-fragmented simple outlines

Again, our analysis describes this data pattern relative to the baseline condition by introducing a new independent variable (MP) and its interactions with previous effects.

Parameters 26–28 only show a significant main effect of MP after presentations 7 (parameter 26, PE = 0.9901, *p* = .0345) and 9 (parameter 26, PE = 2.751, *p* = .0231). The interaction between MP and Symmetry is significant after presentations 3 (parameter 31, PE = 0.74, *p* = .0002), 7 (PE = −1.2584, *p* = .0052), and 9 (PE = −4.1, *p* = .0001).

As can be seen in Figure 4 (row 2, columns 3 vs. 1), the advantage for natural over manmade SP-fragmented simple *asymmetrical* objects is still present for MP-fragments, and the early advantage for manmade over natural SP-fragmented simple *symmetrical* objects is gone for MP-fragments. Also, the advantage of asymmetry for SP-fragmented simple *natural* outlines disappears earlier with MP-fragments, due to the positive interaction effect between symmetry and MP after presentation 3. These observations are partly consistent with prediction 1.

### Patient GA: MP-fragmented simple outlines

Parameters 56–58 show a significant interaction effect between Patient and MP that is positive after presentation 3 and that becomes increasingly negative starting at presentation 5. The interaction effect between Patient, Symmetry, and MP is positive after presentation 1 and becomes bigger starting at presentation 5. As can be seen in Figure 5 (row 2, column 3), GA shows similar behaviour as for SP-fragmented simple outlines.

### Control participants: MP-fragmented complex outlines

Parameters 36–37 show that the interaction effect between MP, Complex, and Manmade is significant after presentation 1 (parameter 36, PE = 0.3601, *p* = .0333), 7 (PE = −0.9301, *p* = .0124), and 9 (PE = −1.3602, *p* = .0039).

From Figure 4 (row 2, columns 4 vs. 2) for *symmetrical* MP complex outlines, we can see the same reversal from an advantage for natural categories after presentation 1 to a disadvantage for natural categories after the third and later presentations, as with symmetrical SP complex outlines. A similar reversal is present for *asymmetrical* complex outlines (SP and MP) but delayed in time, i.e., an advantage for natural categories after presentation 3, and a disadvantage for natural categories after the sixth and later presentations. These observations are consistent with predictions 1 and 2. Note that in general controls perform the worst for symmetrical natural objects: for the latest presentations the survivor probabilities were largest for these outlines regardless of complexity and fragment type.

### Patient GA: MP-fragmented complex outlines

As can be seen in Figure 5 (row 2, column 4), around the third presentation GA starts to show an advantage for manmade over natural objects for both asymmetrical and symmetrical complex outlines, just as for complex SP outlines.

## Discussion

In this study, we used a build-up paradigm where we repeatedly presented a fragmented object outline during a trial, until the observer could correctly name the object depicted at the basic level. We gradually decreased the fragmentation level of the object outlines with each additional presentation in order to maximize the verbal identification performance of patient GA. To investigate the temporal dynamics of category-selective impairments, we tested the effect of object category (natural vs. manmade) and object complexity (simple vs. complex), and statistically controlled for the fragmentation type (straight MPs vs. curved SPs), global symmetry (absent vs. present), the repetition of an object (i.e., first or second trial), and the time of correct responding (i.e., presentation number) using discrete-time survival analysis, and compared GA’s performance to healthy controls.

First, we note that the use of survival analysis was necessary not only to extract dynamic information (see below), but also to get unbiased estimates. For example, the average presentation number for observed correct responses equalled 6 (35% contour) for GA and 2 (12% contour) for the control subjects. However, as can be seen in the survivor functions in Figure 5, for GA the estimated median presentation number for correct naming (the time when *S*(*t*) crosses the line *S*(*t*) = 0.5) was 6 only for asymmetrical manmade objects (and for MP-fragmented, complex, symmetrical manmade object outlines); for natural objects the estimated median presentation number for correct naming was 10 or larger (i.e., half of these objects were still not identified correctly after the tenth presentation). This shows that ignoring right-censored observations before calculating a mean may lead to biased estimates.

Second, regarding the time-course of category-selective impairments, our results mainly confirmed our predictions. Regarding the first prediction, normal controls showed an early advantage for natural over manmade objects in all conditions (except for simple symmetrical outlines, which showed an early disadvantage for natural objects especially with SP-fragments) and this reversed to a late advantage for manmade objects. Furthermore, there was an early disadvantage for symmetry over asymmetry for simple natural objects (again especially with SP-fragments; Figure 4, row 2).

These results are consistent with the idea that the structural representations of early activated candidate object memories in anterior IT can facilitate the grouping of parts into a structural description in posterior IT through top-down feedback from anterior to posterior IT, which will be more useful for structurally similar, i.e., natural objects (Gerlach et al., 2002; 2004; 2006; Panis & Wagemans, 2009). Converging pieces of evidence for this interpretation are: (1) that this early advantage for natural objects is not present for simple symmetrical outlines – top-down facilitation of grouping requires a diagnostic global shape which is not the case for fragmented simple symmetrical outlines and (2) that only simple natural objects show an early disadvantage for symmetry over asymmetry – only the structural descriptions of natural objects are sensitive to global symmetry.

Indeed, it has been suggested that the outline of natural objects (e.g., animals) is more informative for recognition than the outline of artefactual objects (e.g., tools; Lloyd-Jones & Luckhurst, 2002; Riddoch & Humphreys, 2004; Wagemans et al., 2008). Panis, Vangeneugden, Op de Beeck, and Wagemans (2008) found a stronger neuronal sensitivity to the exact shape of (morphed) animal outlines compared to manmade outlines. This could result from the fact that when experiencing objects under natural viewing conditions, the outlines of animals are highly salient because of weak segmentation cues between the parts (e.g., covered with fur) and because animals tend to move against the background in a consistent orientation. In contrast to the more holistic processing of animals, the recognition of tools is thought to rely more on part-based processing because there are strong segmentation cues between the parts and because tools typically do not move and can appear in different configurations and orientations (Riddoch & Humphreys, 2004). In other words, small differences in shape are more relevant for the identification of animals compared to tools. For these reasons, only the structural descriptions of natural objects will be sensitive to global symmetry.

Together with the fact that symmetrical natural objects were identified the slowest, our results suggest that simple symmetrical natural outlines activate the highest number of structurally similar candidate object representations, and therefore take the longest time to identify correctly.

Regarding the second prediction, normal controls indeed show an advantage for manmade over natural objects for later presentations when the fragments are large and correct closure is easy, and this advantage emerges earlier for complex symmetrical than asymmetrical outlines. This relatively late advantage for manmade categories emerges because not grouping but matching is the problem, and natural objects suffer more during the matching process compared to artefactual objects because of their higher structural similarity, given that correct naming has not occurred yet for previous presentations.

Regarding the third prediction, patient GA indeed never showed the early advantage for natural objects (for neither combination of fragment type and complexity) seen in controls. Regarding the fourth prediction, GA indeed showed a relatively late advantage for manmade over natural objects in all conditions (except for simple symmetrical SP-fragmented outlines where the advantage occurred only early, just as for the neurologically intact controls).

These results suggest that GA is especially deficient in top-down guidance from activated shape representations in anterior IT during the grouping of completed parts in posterior IT (shape configuration), and not in grouping small contour segments into longer contours. Only with repeated presentations of a (less and less) fragmented (mainly asymmetrical) outline can GA eventually recognize most manmade objects, possibly by identifying a functionally diagnostic part (see below). The hazard functions for natural objects on the other hand never exceed .15 for GA.

Interestingly, while the control participants correctly identified 50–65% of the objects after the first presentation in a trial (when 10% contour is shown), GA identified only 10% of the objects after the first presentation in a trial, even when they were manmade. GA indeed also shows a slight impairment in naming manmade objects (see Methods) and in object use (Table 1). This is likely due to the lesion in his left frontal lobe, which might also be causing his problems in executive control (Table 1; impaired inhibition of activated but incorrect information). GA shows somewhat better performance for asymmetrical compared to symmetrical manmade objects (see the survivor functions), consistent with the idea that there are less activated candidates when symmetry is absent.

Third, we found a positive effect of repeating an object once across trials (but presented with the other fragment type), which means that objects presented for the first time were identified less frequently than objects that were repeated, regardless of presentation number within a trial. This effect can be interpreted as a classic (conceptual) priming effect (Schacter & Buckner, 1998; Tulving & Schacter, 1990; van Turennout, Bielamowicz, & Martin, 2003).

Fourth, the survival analysis showed that category-selective impairments in identification can change, and even reverse, with increasing waiting time. Such dynamic behaviour is difficult to understand from within the functional/sensory, domain-specific, unitary system, and acquisition accounts. The acquisition account (Gainotti, 2000) could be extended by incorporating the effects of experience with different object categories during early development, next to recurrent processing. Arterberry (2001) discusses how very young children are equally skilled in categorizing living and nonliving objects, and that, with development, increasing knowledge of the function and use of objects, and of the differences between how animate and inanimate objects move (self-propelled vs. other cause, goal-directed vs. goal-less, intentional vs. accidental), may lead to the living-nonliving specialization that is found in adults.

Also, when learning names by supervisors, children might attend different aspects of an object depending on the category it belongs to. For example, when learning about different tools, attention must be directed to one specific part (e.g., screwdriver vs. chisel) and not to its global shape. Gillebert, Op de Beeck, Panis, and Wagemans (2009) showed how object representations are dynamically updated by learning in specific task contexts, by demonstrating that subordinate (but not superordinate) categorization enhances the neural selectivity in inferotemporal cortex for fine shape differences. Thus, although the basic level is the level of abstraction at which objects are optimally distinctive in terms of shape properties (Rosch, Mervis, Gray, Johnson, & Boyes-Braem, 1976), extensive experience with certain object categories can promote a downward shift in the entry-point to more subordinate levels (Tanaka, 2001).

Fifth, the fact that we did not observe strong differences between MP- and SP-fragmented outlines is likely due to the fact that we used long presentation times. We therefore did not observe the early MP advantage for complex objects and the late SP advantage for simple objects as observed by Panis and Wagemans (2009), who used a constant high fragmentation level and very small presentation times that slowly increased across presentations within a trial.

Finally, future studies might focus on four issues. First, instead of using repeated presentations, traditional response time data should be analysed with survival analysis to confirm our interpretations for single presentations (see Panis & Schmidt, 2016). Second, neurally plausible (dynamical) process models should be developed in order to capture the suggested processes quantitatively (Schöner, Spencer, & the DFT research group, 2016). Third, the acquisition account can be tested by creating separate subcategories for food, body-parts, musical instruments, etc. We did not have enough stimuli of each class to make equally large groups. Fourth, the exact consequences of different types of brain damage are currently unknown. For example, it might affect all connections (or cells) or only a subset, or it might generally lower activity or make it more noisy (Lambon Ralph et al., 2007).

In summary, while our results do not exclude a semantic component, they constitute evidence that the category-specific deficit of patient GA is due to a deficit in recurrent processing between the levels of grouping (posterior) and shape description (anterior) in inferotemporal cortex. More generally, our results show the importance of controlling for the time of responding by using discrete time survival analysis when analysing response latencies, regardless of whether they are measured in discrete units (as is done here) or in continuous units (as is typical in RT studies; Panis & Schmidt, 2016).
